# Activated T Cell Trans-Endothelial Migration Relies on Myosin-IIA Contractility for Squeezing the Cell Nucleus through Endothelial Cell Barriers

**DOI:** 10.1371/journal.pone.0075151

**Published:** 2013-09-19

**Authors:** Jordan Jacobelli, Miriam Estin Matthews, Stephanie Chen, Matthew F. Krummel

**Affiliations:** 1 Integrated Department of Immunology, National Jewish Health and University of Colorado Denver, Denver, Colorado, United States of America; 2 Department of Pathology, University of California San Francisco, San Francisco, California, United States of America; IBMC - Institute for Molecular and Cell Biology, Portugal

## Abstract

Following activation, T cells are released from lymph nodes to traffic via the blood to effector sites. The re-entry of these activated T cells into tissues represents a critical step for them to carry out local effector functions. Here we have assessed defects in effector T cells that are acutely depleted in Myosin-IIA (MyoIIA) and show a T cell intrinsic requirement for this motor to facilitate the diapedesis step of extravasation. We show that MyoIIA accumulates at the rear of T cells undergoing trans-endothelial migration. T cells can extend protrusions and project a substantial portion of their cytoplasm through the endothelial wall in the absence of MyoIIA. However, this motor protein plays a crucial role in allowing T cells to complete the movement of their relatively rigid nucleus through the endothelial junctions. *In vivo*, this defect manifests as poor entry into lymph nodes, tumors and into the spinal cord, during tissue-specific autoimmunity, but not the spleen. This suggests that therapeutic targeting of this molecule may allow for differential attenuation of tissue-specific inflammatory responses.

## Introduction

A critical feature of mammalian immune systems is the migration of recently-primed lymphocytes into non-lymphoid organs for the purposes of carrying out effector functions such as killing infected and transformed cells and directed cytokine secretion. To enter tissues, lymphocytes exit the blood flow by extravasating through the vascular endothelial cell wall, a process called trans-endothelial migration (TEM). A class of molecules called chemokines are well understood to serve as chemotactic signals for entry into tissues, with the use of specific chemokines providing further specificity by only attracting certain types of effector cells, based on selective chemokine receptor expression [1]. While this understanding has driven development of chemokine-based therapeutics, relatively less is known about the requisite cytoskeletal remodeling necessary for extravasation and tissue entry. Following selectin- [2] and chemokine- [3] mediated rolling and tethering of lymphocytes within tissue vasculature, to extravasate lymphocytes must transmigrate through the endothelial cell vascular wall.

The general mechanism of leukocyte interstitial migration within tissue requires new actin polymerization in the leading edge of cells, a region termed the pseudopod, which effectively extends the cytoplasm into new regions of the tissue. Integrin-dependent or independent contacts formed with the underlying substrata can then either allow leukocytes to pull themselves along the stroma [4-6] or, in concert with additional adhesions formed along the three-dimensional surface of the migrating cell, to effectively push themselves through the tissue (a motility mode termed ‘walking’) [5,7]. Myosin-IIA (MyoIIA) generally accumulates at the back of these adhesions [7,8] and is required to lift up the adhesion as the cell translocates and extends the adhesion forward. T cells in which MyoIIA is pharmacologically blocked can thus display extremely elongated trailing edge ‘uropods’ [8,9].

In the case of T cells that form multiple contact zones with the substrate for the purpose of ‘walking’ motility, MyoIIA clusters were observed to surround adhesion zones as they were radially extinguished [7]. This form of motility with multiple adhesions appears to be completely dependent upon MyoIIA. Mouse T cells express a single Myosin-II isoform, MyoIIA/MyH9 [10], and we recently described a T cell-conditional knockout mouse strain with defects in interstitial lymph node migration associated with over-adhesion [5]. Multiple lines of evidence support that lymphocytes can still migrate in the absence of MyoIIA via continuous adhesions, albeit more slowly and with the potential for forming elongated uropods that do not efficiently de-adhere [5,7-9].

During extravasation, subsequent to rolling and firm-adhesion, T cells crawl over endothelial cells to find permissive extravasation sites and then undergo diapedesis, the final step of TEM through the endothelial barrier [11,12]. TEM involves multiple and unique stages of force generation and requires penetration of the T cell body through small openings in the endothelial barrier, either through opening of an endothelial cell-cell junction (paracellular TEM) or by tunneling through an endothelial cell (transcellular TEM) [13,14]. MyoIIA-mediated force generation can be expected to play roles during various stages of TEM, particularly during extravasation into non-lymphoid tissues that are not commonly sites of lymphocyte entry and may possess more stringent endothelial barrier properties.

How MyoIIA affects each step of the TEM process has recently started to be elucidated. In genetic knockouts, we observed that naïve MyoIIA null T cells were numerously adhered to high endothelial venules (HEVs) in lymph nodes and showed over-adherence defects in additional assays [5]. Similarly, using transient treatment of naïve T cells with a Myosin-II–blocking drug, it has been proposed that T cells require this motor to complete transmigration of the rigid nucleus during TEM [15]. Such a model was also proposed for the movement of dendritic cells through dense collagen matrices [4]. However, our data on homeostatic naïve T cell trafficking suggested that the dominant long-term effect of MyoIIA deficiency in resting T cells manifested at the level of lymph node retention, presumably due to defects in migration to the efferent lymphatics [5]. How MyoIIA regulates TEM and trafficking of effector T cells remains largely unknown.

Here, we use genetic deletion of MyoIIA in activated T cells to examine the role of this motor protein for entry of effector T cells into both lymphoid and non-lymphoid tissues under homeostatic and inflammatory conditions. We demonstrate the capability of these MyoIIA-deficient T cells to thread leading edge material across endothelial barriers but a marked defect in completing nuclear translocation during diapedesis. Positioning of MyoIIA in T cells observed in the act of transmigrating shows a distribution of this motor protein around and behind the nucleus, consistent with a role for MyoIIA in squeezing nuclei through restrictive openings. Overall, our data show that inhibition of this motor protein significantly reduced effector T cell TEM and entry into tissues.

## Results

**Figure 1:**
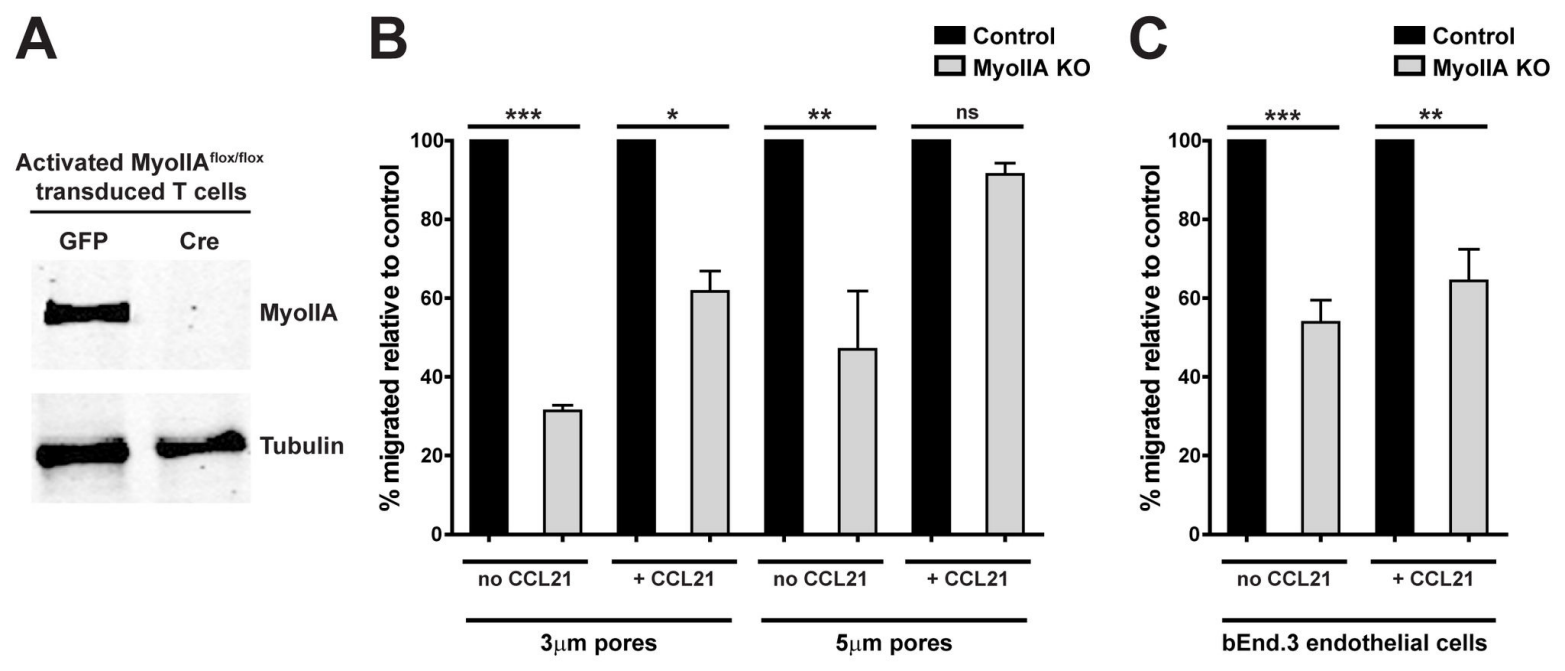
Transwell migration defects of activated MyoIIA-deficient T cells. T cells from MyoIIA^flox/flox^ mice were activated and then retrovirally transduced with either Cre-GFP (MyoIIA KO) or GFP only (control). T cells were then sorted for GFP^+^ cells 48-72h post-transduction. Fluorescently-labeled sorted control and MyoIIA KO T cells were mixed at a 1:1 ratio and used for experiments. A) Representative blot of MyoIIA KO in the Cre-transduced T cells vs. GFP-transduced control T cells. Tubulin expression levels in the same samples are shown as loading controls. B) Percent migration through 3μm or 5μm pore transwells of control and MyoIIA KO T cells in the absence or presence of 1μg/ml CCL21 in the lower wells. Data are the mean (±SEM) of 2 independent experiments. C) Percent transwell trans-endothelial migration of control and MyoIIA KO T cells through a bEnd.3 brain endothelial cell monolayer seeded on 5μm pore transwells in the absence or presence of 1μg/ml CCL21 in the lower wells. Data are the mean (±SEM) of 3 independent experiments. * p<0.05, ** p<0.01, and *** p<0.001.

We and others have shown that MyoIIA plays an important role in naïve T cell trafficking and TEM [5,15]. To reach inflamed tissues and carry out their functions effector T cells must also extravasate from the blood circulation by TEM. However, as opposed to the vasculature within lymph nodes, extravasation into non-lymphoid tissues may have to overcome tighter endothelial barriers for T cell entry. Based on their size and activation state we thought that activated T cells might require greater force generation during entry into tissues and thus be especially sensitive to the loss of MyoIIA activity compared to naïve T cells. Therefore, we sought to determine the contribution of MyoIIA to activated T cell extravasation. We have established a line of transgenic mice in which MyoIIA can be conditionally knocked-out in T cells using the Cre-loxP system [5]. However, MyoIIA plays a role in the cytokinesis step of cell division [16] and T cells lacking MyoIIA function have proliferation defects, often resulting in multi-nucleated cells and poor viability (J.J. and M.F.K. unpublished data). We therefore devised an experimental system to deplete MyoIIA in activated T cells without significantly affecting their viability. To this end, T cells from MyoIIA^flox/flox^ mice [5] were *in vitro* activated and then, after the T cells were activated and had started proliferating, the T cells were transduced with a retroviral vector encoding Cre-GFP to genetically eliminate MyoIIA expression. As controls we used activated T cells derived from the same MyoIIA^flox/flox^ mice transduced with a GFP-only retroviral vector. With this system, MyoIIA depletion (MyoIIA KO) occurred over the following 72h, allowing T cells to proliferate while minimizing effects on viability. At this point, T cells were ‘activated’ and yet contained no detectable, or only minimal, MyoIIA compared to control T cells (typical result shown in Figure 1A).

We initially tested migration of activated MyoIIA KO T cells in ‘transwell’ assays through membranes with different pore sizes. Loss of MyoIIA in activated T cells resulted in reduced transwell migration, particularly through tight 3μm pores (Figure 1B). The presence of chemokine mitigated this migration defect but only during migration through more permissive 5μm pores (Figure 1B). Given that the inhibition was most prominent when T cells were challenged with small 3μm pores, as compared to larger 5μm pores, this suggested that force generation via MyoIIA was required to squeeze T cells through restrictive barriers. We also tested migration through 5μm pore transwell membranes overlaid with a monolayer of brain-derived bEnd.3 endothelial cells and saw a significant reduction in migration regardless of the presence or absence of chemokine (Figure 1C). These results supported the view that MyoIIA is not strictly necessary for chemokine sensing or for directional migration under these model settings, but instead may be involved in facilitating the squeezing of T cells through restrictive barriers.

**Figure 2:**
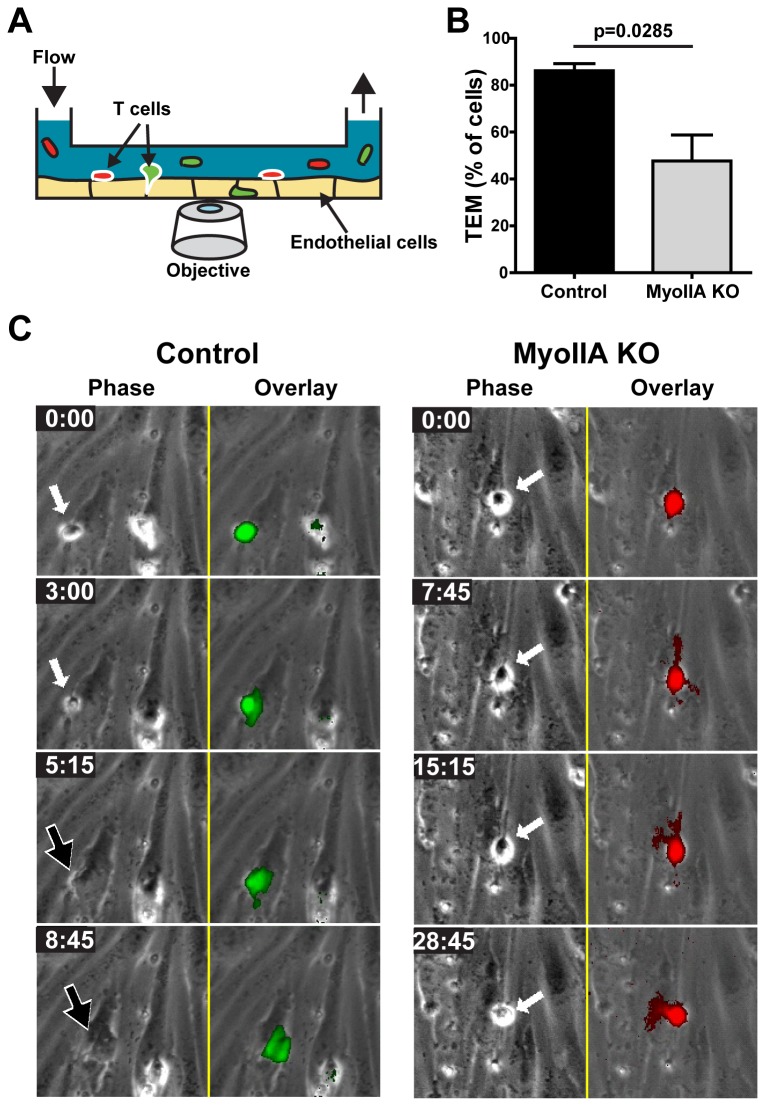
MyoIIA deficiency in activated T cells causes defects in trans-endothelial migration (TEM) under flow. Control and MyoIIA KO activated T cells were fluorescently labeled, mixed at a 1:1 ratio and perfused into a flow chamber containing a monolayer of bEnd.3 brain endothelial cells and then kept under a physiological shear flow rate of 2 dyne/cm^2^ for 30 min. During this time, phase contrast, green and red fluorescence images were acquired every 15 sec. A) Schematic showing the TEM flow chamber setup. B) Percentage of transmigrating T cells, calculated by quantifying the number of T cells that lost their phase contrast ring and underwent a stepwise darkening in the phase contrast channel during the time-lapse, relative to the total number of T cells adhered to the endothelial cell monolayer. Data are the mean (±SEM) of 3 independent experiments. C) Selected time-point images of representative control and a MyoIIA KO T cells during TEM. Phase contrast images (left) and fluorescence overlay on the phase contrast (right) are shown. White arrows point to T cells above the endothelial monolayer; black arrows point to T cells that have completed TEM and are under the endothelial monolayer. Time in min: sec.

**Figure 3:**
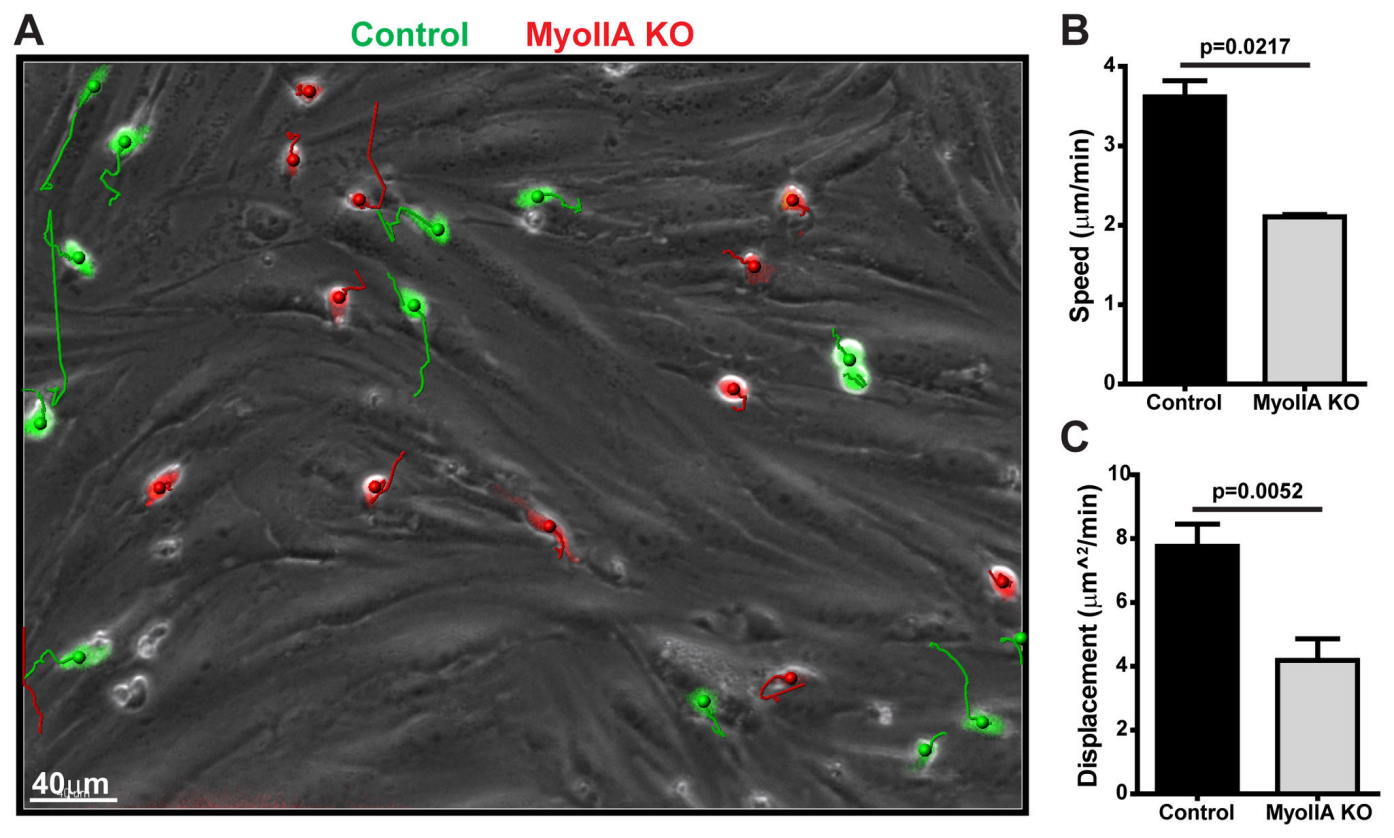
Lack of Myosin-IIA impairs T cell motility on endothelial monolayers. Fluorescently-labeled control (green) and MyoIIA KO (red) T cells were perfused onto endothelial monolayers under flow and imaged by time-lapse fluorescence microscopy. A) Representative image of fluorescence overlay (green and red) on phase contrast image (grey) from the analysis of a 15 min time-lapse imaging of T cell crawling and TEM. The color-coded tracks show the migration paths of the T cells during the time-lapse. Scale bar = 40μm. B) Speed of control and MyoIIA KO T cells crawling on endothelial cell monolayers. Data are the average (±SEM) of the mean of 3 independent experiments. C) Displacement (shown as distance^2^/time) of control and MyoIIA KO T cells crawling on endothelial cell monolayers. Data are the average (±SEM) of the mean of 3 independent experiments.

We next set up an *in vitro* system to more closely recapitulate TEM under physiological shear flow (Figure 2A), and more precisely determine which steps of TEM rely on MyoIIA-generated mechanical force. We analyzed TEM under flow of activated T cells through a monolayer of brain-derived endothelial cells in real-time, using phase contrast microscopy (Movies S1-S3). Quantification of this data showed that MyoIIA KO T cells had a ~50% reduction in the ability to complete TEM relative to control T cells (Figure 2B). Our imaging data revealed that although activated MyoIIA KO T cells were able to adhere to the endothelial monolayer and initiate TEM by inserting pseudopodal projections underneath the endothelial cells, they were defective in completing TEM (Figure 2C and Movie S3). This was in contrast to control T cells, the majority of which readily completed TEM (Figure 2C and Movie S2). Typically, in the MyoIIA KO T cells that attempted but did not complete TEM, the main portion of the cell body remained above the endothelial cell monolayer, as evidenced by the permanence of the phase contrast ring around these cells. We also analyzed the crawling behavior of control and MyoIIA KO activated T cells on endothelial cells and found that relative to controls, both the speed and displacement of MyoIIA KO T cells was significantly reduced (Figure 3 and Movie S4). This could be in part due to the MyoIIA KO T cells getting stuck during TEM, and in part to the known defects in crawling of MyoIIA-deficient T cells, both on two-dimensional surfaces as well as in confined three-dimensional environments [5,7-9].

**Figure 4:**
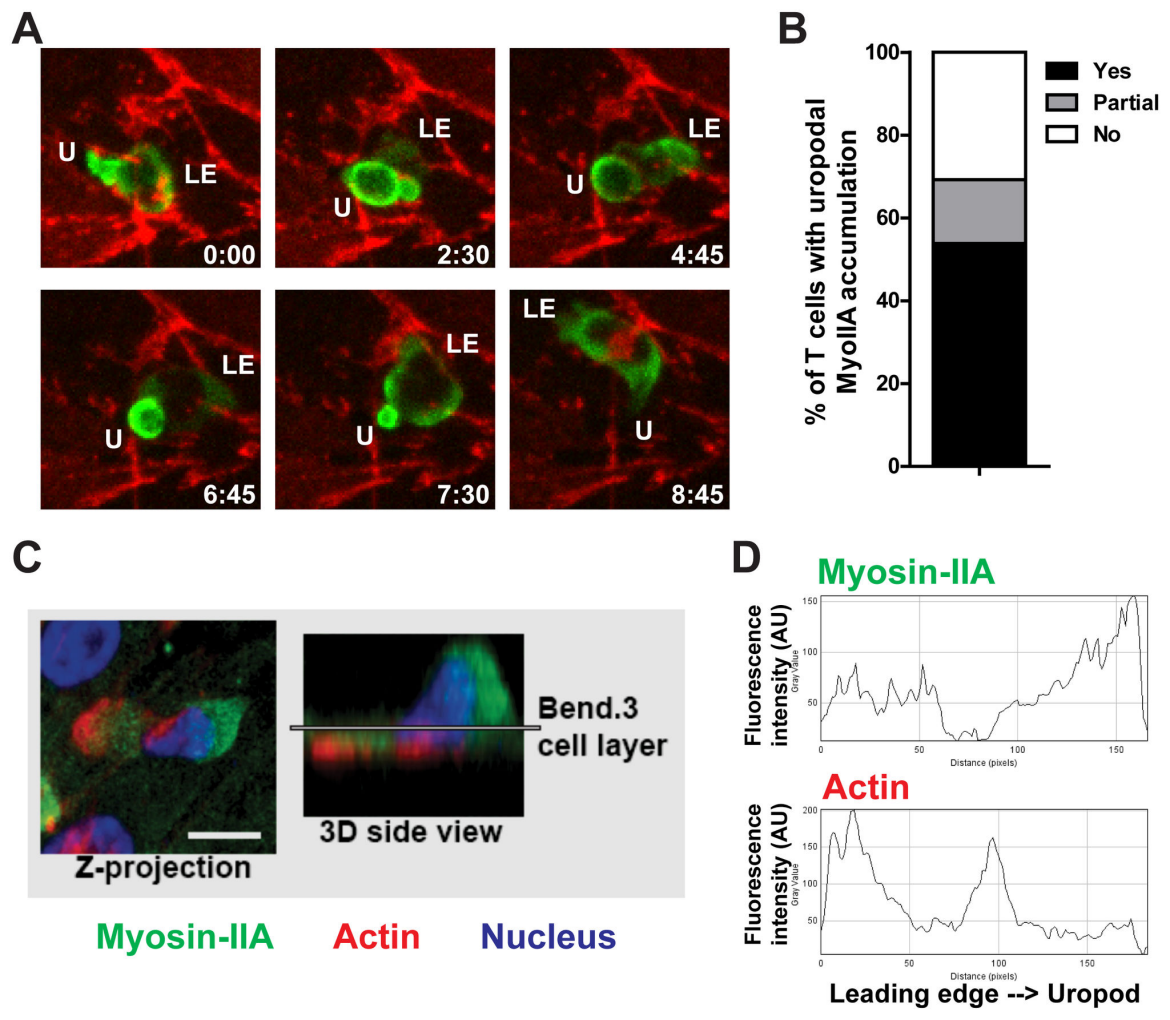
MyoIIA is enriched at the uropod of transmigrating T cells. A) Activated T cells expressing a fusion protein of GFP and MyoIIA were imaged by time-lapse confocal microscopy while undergoing TEM under flow. Representative maximum Z-projection images of selected time-points of a T cell expressing GFP-MyoIIA (green) undergoing TEM. The endothelial cell monolayer was stained with anti-CD31 (red) to visualize endothelial cell junctions. B) Percentage of T cells with GFP-MyoIIA enrichment at the uropod while undergoing TEM. Data are pooled from 2 independent experiments imaging GFP-MyoIIA expressing T cells undergoing TEM by time-lapse microscopy. C) Example maximal Z-projection image and 3D reconstruction of the side view of a transmigrating T cell stained for MyoIIA, filamentous actin, and nucleus. WT activated T cells were perfused under physiological shear flow onto an endothelial cell monolayer and fixed with paraformaldehyde 15 min later. The cells were then fluorescently stained and 1μm thick Z-stacks were acquired with a spinning-disk confocal microscope. The endothelial cell monolayer position in the Z-stack is indicated by the line in the 3D side view and was identified by the presence of a network of actin stress fibers (not shown). D) Quantification of fluorescence intensity of MyoIIA and filamentous actin along the front to back axis of the transmigrating T cell shown in C.

Given the significant reduction of TEM in MyoIIA KO activated T cells, we wanted to gain further insight into the mechanism of MyoIIA function in this process. Depending on the experimental system, lack of MyoIIA in activated T cells can result in inefficient de-adhesion leading to elongated uropods [9,16] or increased spreading [7,15]. In addition, MyoIIA contractility plays an important role in facilitating leukocyte migration through dense collagen matrices [4,7], possibly by helping to squeeze the rigid nucleus through constrictive openings. As a way to understand the possible function of MyoIIA in TEM, we determined the distribution of this myosin motor protein in activated T cells undergoing TEM under flow using both real-time imaging and immuno-fluorescent staining. For real-time imaging of MyoIIA during TEM we used T cells derived from transgenic mice that express a GFP-MyoIIA fusion protein [17]. Our imaging data showed an accumulation of GFP-MyoIIA at the uropod of transmigrating T cells, with more than two thirds of T cells showing a full or partial enrichment in the uropod during the diapedesis step (Figure 4A-B). This enrichment tended to reach maximal levels just as the T cell was squeezing through the endothelial monolayer (Figure 4A and Movie S5). Immuno-fluorescent staining of wild-type T cells confirmed the distribution of MyoIIA at the back of T cells during TEM (Figure 4C-D). With this analysis we also determined the distribution of MyoIIA relative to sites of actin remodeling and the nucleus in T cells undergoing TEM. Our data showed that MyoIIA is often found cradling the nucleus of transmigrating T cells (Figure 4C). This suggested that one of the mechanisms by which MyoIIA promotes TEM may be squeezing of the nucleus through the endothelial cell monolayer.

**Figure 5:**
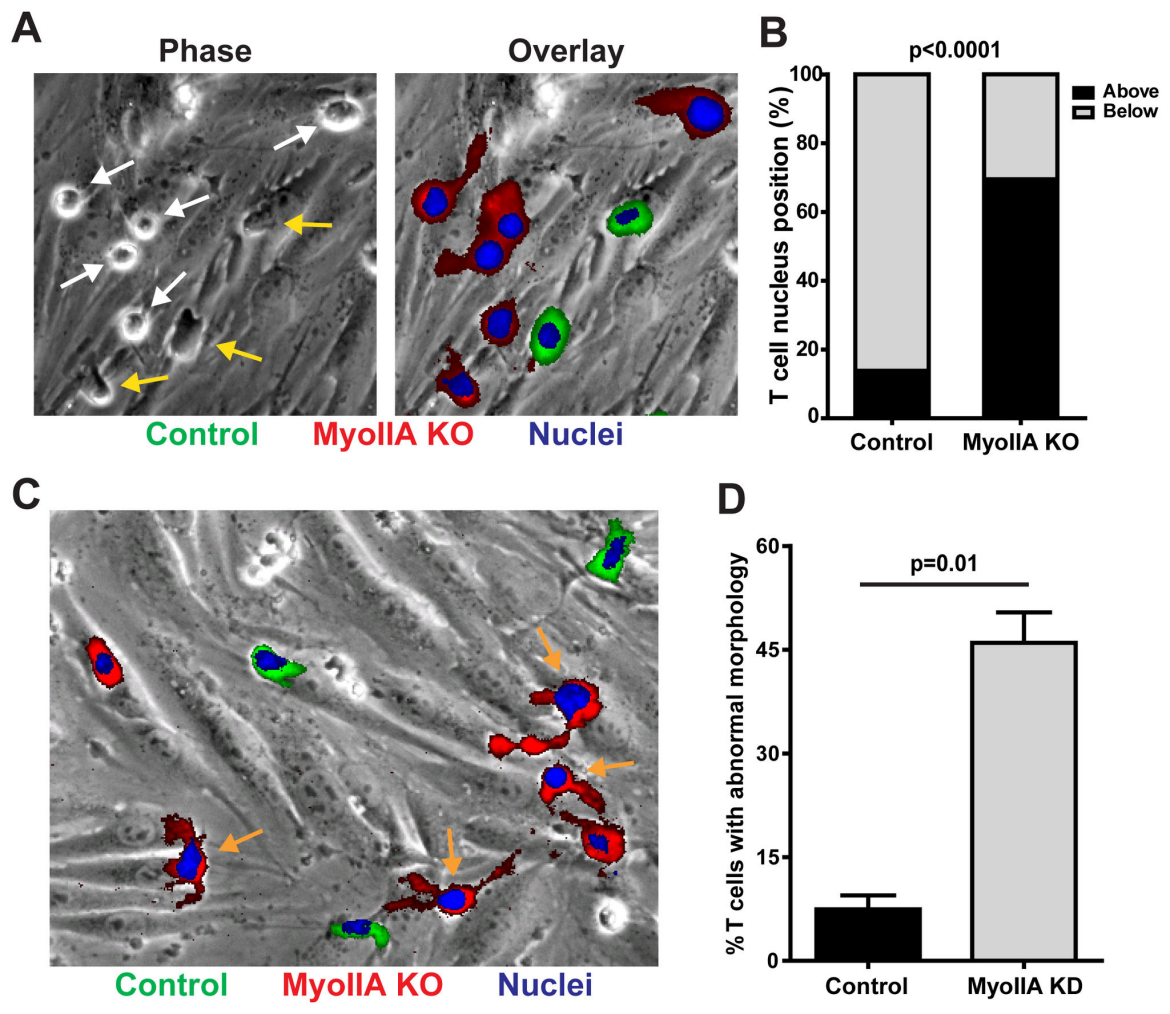
MyoIIA facilitates TEM by squeezing the T cell nucleus through the endothelial cell monolayer. A) MyoIIA KO T cells are impaired in squeezing their nuclei through the endothelial cell monolayer to complete TEM. Control (green) and MyoIIA KO (red) T cells were also labeled with the vital nuclear dye Hoechst (blue) and the position of the nucleus relative to the T cell body and endothelial cell monolayer during TEM was imaged 20 min after addition of the T cells to the endothelial cell monolayer. Representative phase contrast image (left) and fluorescence overlay on the phase contrast (right) are shown. White arrows indicate T cells with nuclei still above the endothelial cell monolayer; yellow arrows indicate T cells with nuclei underneath the endothelial monolayer. B) Quantification of control and MyoIIA KO T cells with their nuclei above or below the endothelial cell monolayer during TEM, analyzed 20 min after addition of T cells to the endothelial monolayer. Data are pooled from 2 independent experiments. Fisher’s exact test yields a p<0.0001. C) MyoIIA KO T cells have abnormal elongated morphology and multiple protrusions during TEM. Control (green) and MyoIIA KO (red) T cells were also labeled with the vital nuclear dye Hoechst (blue) and perfused onto endothelial monolayers under physiological shear flow and imaged by fluorescence microscopy 20 min later. Representative image of fluorescence overlay on the phase contrast image. Orange arrows indicate abnormal T cells with elongated protrusions and/or multiple protrusions. D) Quantification of control and MyoIIA KO T cells with abnormal morphology during TEM. Data are the mean (±SEM) of 3 independent experiments.

We next analyzed the position of the nucleus in control and MyoIIA KO activated T cells undergoing TEM. This analysis revealed that, after 20 min of incubation on the endothelial monolayer under flow, less than 15% of control T cells had their nuclei above the endothelial cells, while almost 70% of MyoIIA KO T cells had nuclei still above the endothelial monolayer (Figure 5 A-B). This further indicated that, while control T cells rapidly completed TEM, MyoIIA KO T cells did not efficiently squeeze their rigid nucleus through the endothelial monolayer and remained stuck during the process. We then analyzed the morphology of activated T cells undergoing TEM. We found that, relative to control T cells, a significantly higher portion of MyoIIA KO T cells had abnormal elongated morphologies with multiple and long protrusions (Figure 5 C-D). These elongated MyoIIA KO T cells did not appear to be caused by defects in uropod retraction, since the T cell body was typically still above the endothelial monolayer, but rather by the generation of protrusions during the attempt to undergo TEM, with many MyoIIA KO T cells having multiple protrusions (Figure 5C).

**Figure 6:**
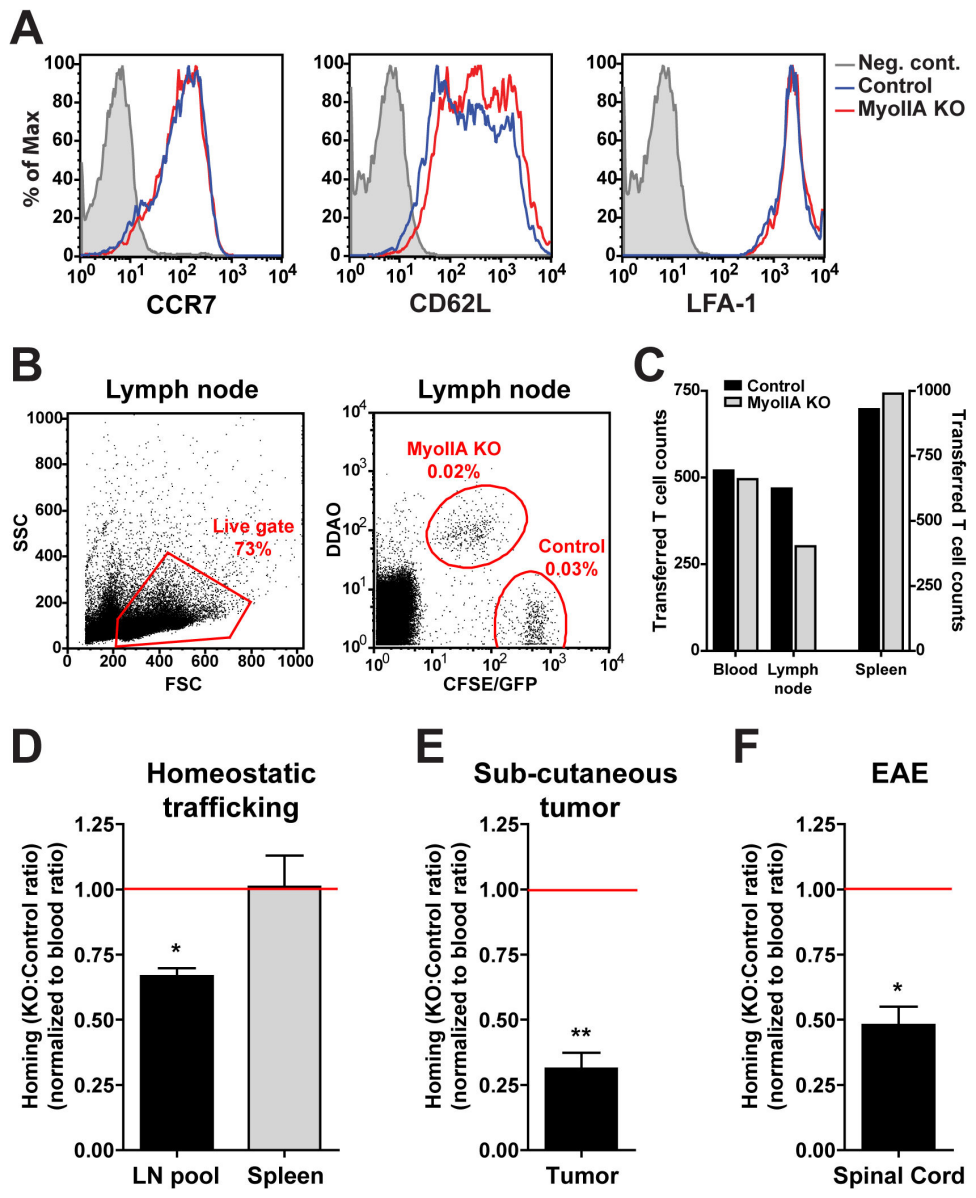
MyoIIA deficiency in activated T cells causes homing defects during homeostatic and inflammation-driven trafficking. A) Chemokine receptor and adhesion molecules are similarly expressed on control and MyoIIA KO activated T cells. Flow cytometry expression profile of CCR7, CD62L and LFA-1 of control and MyoIIA KO activated T cells. Data in A is representative of 2 independent experiments. B-C) Representative examples of flow cytometry analysis and quantification of transferred T cell homing *in* *vivo*. Control and MyoIIA KO T cells were labeled with different fluorescent dyes and transferred intravenously into recipient mice, 18h post-transfer tissues were harvested and the number of transferred cells was quantified. Examples of transferred T cell flow cytometry gating (B) and enumeration (C) are shown. D-F) Reduced tissue homing of MyoIIA KO activated T cells *in* *vivo*. The ratio of intravenously transferred MyoIIA KO vs. control T cells from dissociated tissues was quantified by flow cytometry and normalized to the ratio in the blood. A ratio below 1.0 indicates impaired entry of MyoIIA KO cells into the indicated tissue. D) Quantification of MyoIIA KO activated T cell homing to lymph nodes and spleen of untreated recipient mice 18h post-transfer. Data in D are the mean (±SEM) of 4 independent experiments. E) Homing of MyoIIA KO T cells to ectopic sub-cutaneous tumors 18h after intravenous transfer, quantified by flow cytometry. Data in E are the mean (±SEM) of 3 independent experiments. F) Reduced homing of MyoIIA KO T cells to the spinal cord of recipient mice with ongoing EAE 24h after intravenous transfer. Data in F are the mean (±SEM) of 3 independent experiments. * p<0.05 and ** p<0.01.

Finally, we wanted to determine whether the observed *in vitro* TEM defects of activated MyoIIA KO T cells would affect their ability to traffic and enter tissues *in vivo*. First we determined if MyoIIA depletion affected the expression of chemokine receptor and adhesion molecules, which could alter their trafficking potential. Flow cytometry analysis of control and MyoIIA KO activated T cells showed that the chemokine receptor CCR7, the selectin CD62L, and the integrin LFA-1, were all expressed at levels comparable to control cells (Figure 6A). This ruled-out that potential *in vivo* trafficking phenotypes of MyoIIA KO T cells would be due to altered expression of these surface molecules.

Next, we determined the extravasation potential of MyoIIA KO activated T cells versus control activated T cells into different tissues under homeostatic and inflammatory conditions. First we assessed homeostatic trafficking to lymph nodes and spleen. For these experiments, we intravenously transferred a 1:1 ratio of control and MyoIIA KO T cells, fluorescently labeled with different dyes, into unimmunized wild-type recipient mice and then quantified the number of transferred cells in different tissues by flow cytometry (Figure 6B and C). Quantification of the number and ratio of MyoIIA KO versus control activated T cells in lymph nodes and spleen 18 hours after adoptive transfer showed a ~34% reduction in the number of MyoIIA KO activated T cells that had homed to the lymph nodes (Figure 6D). Interestingly, this activated T cell trafficking result was the opposite of what we previously observed with naïve T cells [5], and suggested that in activated T cells the major phenotype caused by loss of MyoIIA manifested itself at the level of TEM and lymph node entry rather than lymph node retention and egress.

As a further control for the *in vivo* homeostatic trafficking experiments we also repeated these experiments using control cells obtained by transducing T cells from MyoIIA^wt/wt^ mice with Cre-GFP (rather than MyoIIA^flox/flox^ T cells transduced with a GFP only retroviral vector) and obtained a similar defective lymph node homing phenotype of MyoIIA KO T cells compared to control cells (data not shown).

Based on these findings, we then tested the trafficking of MyoIIA KO activated T cells into non-lymphoid tissues under inflammatory conditions in a tumor model and in an autoimmune setting. First, we measured trafficking of Ovalbumin (Ova)-specific activated CD8 T cells to the site of ectopic sub-cutaneous tumors expressing Ova. Our data showed that under these circumstances MyoIIA played an important role in trafficking of activated T cells to an inflammatory site, with a close to 3-fold reduction in homing to the tumor site (Figure 6E).

Finally, we wanted to determine if MyoIIA could modulate the ability of activated self-reactive T cells to reach their target organ and if MyoIIA contractility played an important role in breaching the restrictive endothelial blood-Central Nervous System barrier. For these experiments, Myelin Oligodendrocyte Glycoprotein (MOG)-specific MyoIIA^flox/flox^ CD4 T cells were *in vitro* activated and transduced with either Cre-GFP (MyoIIA KO) or GFP only (control) and transferred into recipient mice with ongoing Experimental Autoimmune Encephalomyelitis (EAE). 24 hours after adoptive transfer, the number of MOG-specific control and MyoIIA KO T cells that had infiltrated the spinal cord of recipient EAE mice was quantified. Our results indicated that loss of MyoIIA function significantly reduced the ability of self-reactive T cells to extravasate through the spinal cord vasculature and infiltrate the Central Nervous System (Figure 6F).

## Discussion

Prior to this work it was not clear how effector T cells would be influenced by modulation of force generation and transmission within T cells during TEM. Our data strongly points to the final step of TEM and tissue entry as being highly reliant on intrinsic T cell force transmission. As indicated by our TEM studies, in the absence of MyoIIA function T cells can effectively generate cytoplasm protrusions through the endothelial wall. However, MyoIIA-deficient T cells fail to efficiently complete TEM. This phenotype, in addition to the subsequent poor interstitial migration of those T cells that do complete TEM [5], will combine to likely limit effector T cell function in peripheral tissues. Although it was not examined in this report, it could also be possible that T cells lacking MyoIIA that are stuck halfway through the vascular barrier, unable to complete TEM or retract their protrusion and return to the blood flow, would ultimately die due to the effects of ongoing shear forces.

After adhering to the endothelial vascular wall, T cells migrate along the luminal surface of the endothelium. It is believed that this intra-luminal crawling is a way to search for permissive extravasation sites. As they crawl over endothelial cells, T cells form filopodia protrusions [18], which may aid in sensing of chemokines on the endothelium and guide T cells to extravasation sites. Eventually a subset of these filopodia can grow into larger protrusions that invade the endothelium to initiate diapedesis [18], potentially in response to ‘hot spots’ of chemokine release from the endothelium [19]. In addition, during transcellular TEM a different membrane protrusion, the invasive podosome, has been described as being critical to diapedesis [13]. In both cases, force generation and transmission are critical to allow extension of these protrusions and pushing of the T cell cytoplasm with its content through small openings in the endothelial barrier [12]. Actin polymerization and Myosin-II contractility can generate the mechanical forces necessary for extravasation [12]; however, the exact mechanisms of action of the T cell actin-myosin cytoskeleton during TEM remain largely unclear.

How does MyoIIA-generated mechanical force facilitate TEM completion? A potential way that MyoIIA can affect the overall TEM process is by modulating T cell motility over endothelial cells (through adhesion and de-adhesion mechanisms on this substrate), this may then affect the T cell pathfinding ability to reach permissive TEM sites. However, our observation that squeezing of the T cell nucleus through the endothelial barrier is the key step that is defective in the absence of MyoIIA suggests that a major function of MyoIIA-generated force is to enable efficient propulsion of the nucleus through endothelial openings. In support of this mechanism, we show that in T cells caught in the act of TEM, MyoIIA is enriched within the region surrounding the not-yet-transmigrated nucleus. Furthermore, Stein and colleagues also support the concept that, while de-adhesion is important for migration, nucleus squeezing during TEM is the major bottleneck for T cells in the absence of MyoIIA function [15]. Stein and colleagues suggest that diapedesis is intercellular adhesion molecule (ICAM)-independent [15,20]. If this is the case, this would suggest that force is transmitted from the T cell onto a fixed surface of the endothelium via other adhesion molecules. An alternative mechanism is that MyoIIA squeezes the cytoplasm and nucleus from within the T cell, via shape deformation, in the absence of direct force transmission to the surrounding endothelium. MyoIIA contractility could also conceivably promote the equivalent of an expanding membrane ring during diapedesis and generate force outward to open up space in the endothelial junction through which the T cell nucleus can pass.

In addition to force transmission mediated by T cells during TEM, the endothelium is also likely involved in transmitting mechanical force to facilitate TEM. Indeed, a recent *in vitro* study has been interpreted as showing that Myosin-II activity is necessary within the endothelial cells in order to exert force to pull or push T cells through the endothelium [21]. With this in mind, we focused our studies on the role of lymphocyte Myosin-II in force generation during TEM. Our study does not exclude the possibility that endothelial force generation through Myosin-II may be important, but clearly shows that T cell intrinsic Myosin-II function is also required.

A recent paper studying human effector memory T cells has also identified a requirement for MyoIIA in TEM. However, in that study MyoIIA was required for T cell receptor (TCR)-driven TEM but surprisingly dispensable for chemokine-driven TEM [22]. This difference compared to our findings and those of Soriano et al. [15] may be due to the different model systems used such as human vs. mouse cells, and the types of T cells and endothelial cells.

A growing body of work has been accumulating showing that force transmission by MyoIIA in naïve T cells is important for entry into lymph nodes during recirculation, largely shown by adoptive transfer experiments [5,15]. Intriguingly, however, using genetic ablation of MyoIIA we showed that the long-term effect of MyoIIA loss in naïve T cells is primarily to lower their interstitial motility rate and to restrict their steady-state egress from lymph nodes [5]. Here we report that activated T cells also rely on MyoIIA for TEM and homing to sites of inflammation. Thus, a chronic inhibition of MyoIIA-mediated contractility would be expected to be potentially immunosuppressive for lack of overall antigen surveillance and effector functions by T cells. The sum of these findings suggests that, were it possible to direct class-II myosin specific interfering RNAs or chemical inhibitors (such as blebbistatin [16]) exclusively to the T cell compartment, this might generate useful forms of immunosuppression. Lack of effective surveillance and recirculation of T cells could reduce reactivity for rare antigens, while reduced entry into peripheral organs (such as the spinal cord) may attenuate tissue-specific autoimmune responses. On the other hand, since homing to the spleen is minimally affected by MyoIIA inhibition, surveillance of blood-borne pathogens may remain functional. However, potential unwanted side-effects of MyoIIA inhibition in the T cell compartment would have to be more closely studied.

## Materials and Methods

### Ethics statement

All experiments involving mice were approved by the Institutional Animal Care and Use Committees of the University of California, San Francisco (Protocol #AN084275) and National Jewish Health (Protocol #AS2811-02-14). All efforts were made to minimize mouse suffering.

### Mice

Wild-type (WT) C57BL/6 (cat. #000664) mice and 129S1 x C57BL/6 F1 mice (cat. #101043) were purchased from Jackson Laboratory. Mice containing loxP flanked (‘floxed’) alleles of the MyoIIA heavy-chain (Myh9) were generated as described in [5]. To obtain self-reactive CD4 T cells for EAE trafficking experiments, MyoIIA^flox/flox^ mice were crossed with mice expressing a TCR specific for the self-antigen MOG [23] (purchased from Jackson Laboratory). To obtain Ova-specific CD8 T cells, MyoIIA^flox/flox^ mice were crossed with mice expressing an Ova-specific TCR (OT1 TCR mice, purchased from Jackson Laboratory). Mice expressing GFP fused to the N-terminus of the heavy chain of MyoIIA were generated by Zhang et al. [17] (kindly provided by Dr. Robert Adelstein, NHLBI, Bethesda, MD).

### Cells

T cells were cultured using RPMI 1640 with the addition of L-glutamine, penicillin, streptomycin and β-mercaptoethanol (all purchased from Invitrogen). Activated/effector T cells were obtained by harvesting lymph nodes and spleen from donor mice and activating T cells *in vitro* with plate-bound CD3 antibodies (BioXcell) and soluble CD28 antibodies (BioXcell) for 2 days. The activated T cells were then removed from the antibody coated plates and grown in culture in complete RPMI with the addition of 10U/ml of recombinant Interlukin-2 [24] (IL2, obtained through the AIDS Research and Reference Reagent Program, Division of AIDS, NIAID, NIH from Dr. Maurice Gately, Hoffmann - La Roche Inc.). In the case of non-TCR transgenic donor mice, the resulting population of activated T cells was a mix of CD4^+^ and CD8^+^ T cells. The bEnd.3 brain endothelial cell line (purchased from ATCC) was cultured in complete DMEM (Invitrogen). The Ova-expressing EL4 lymphoma cell line (EG.7-Ova, obtained from ATCC) was grown in RPMI 1640.

### T cell retroviral transduction

Activated MyoIIA KO T cells were obtained by transduction of T cells from MyoIIA^flox/flox^ mice with retroviruses coding for either Cre-GFP (to eliminate MyoIIA expression) or GFP only (as a control) 48h after initial *in vitro* activation. The Cre-GFP and GFP retroviral vectors were kindly provided by Dr. Benjamin Braun (UCSF, San Francisco, CA). 48-72h post-transduction GFP^+^ T cells were sorted and used for experiments; depletion of MyoIIA in the Cre-transduced T cells was routinely verified by western blotting or intra-cellular staining and FACS analysis similarly to the methods described in [5]. For western blotting analysis we used Enhanced Chemiluminescence detection (GE Healthcare Life Sciences) or the Odyssey near-infrared imaging system (Li-cor Biosciences). Control T cells were obtained by transducing MyoIIA^flox/flox^ with GFP only retroviruses or alternatively in some experiments MyoIIA^wt/wt^ T cells were transduced with Cre-GFP retroviruses.

### Transwell migration assay

Sorted activated control and MyoIIA KO T cells were further differentially labeled with either 1-2μM Carboxy-fluorescein diacetate succinimidyl ester (CFSE) (Invitrogen) or 10μM CellTracker Orange CMTMR (Invitrogen) and mixed at a 1:1 ratio and used for transwell migration assays. Between experimental repeats, fluorescent labels were swapped between control and MyoIIA KO cells to control for potential effects of specific dyes. 0.5-1x10^6^ labeled T cells in RPMI supplemented with 1% fatty acid-free BSA (Calbiochem) and 10 mM HEPES were added to the top chambers of Transwell plates (Corning) and allowed to migrate for 3h at 37°C in the presence or absence of chemokine in the lower well. Each condition was set up in duplicate transwells. Migrated T cells were then collected from the bottom wells and quantified for a fixed period of time (2-3 min) using a flow cytometer (FACS Calibur, BD Biosciences, or a CyAn ADP, Beckman Coulter). Known amount of cells, acquired for the same time, were used as standards to determine the number of migrated cells.

### Trans-endothelial migration under flow

Sorted activated control and MyoIIA KO T cells were further differentially labeled with either 1μM CFSE or 2μM CellTracker Orange CMTMR and mixed at a 1:1 ratio, and then perfused into a flow chamber (μ-slide VI, IBIDI) coated with a monolayer of bEnd.3 endothelial cells. T cells were resuspended at 2x10^6^ cells/ml and initially perfused in the flow chamber at 0.25 dyne/cm^2^ for 5 min, the flow was then raised to 2 dyne/cm^2^ (physiological shear flow) for the remainder of the imaging. Phase contrast and fluorescence images were acquired every 15 sec for 30 min. T cells that lost the white phase contrast ring and underwent a stepwise darkening in the phase contrast channel during the timelapse were scored as transmigrating cells. In a subset of experiments T cells were labeled with the nuclear dye Hoechst and transmigration was also determined by acquiring Z-stacks and determining the position of the cell body containing the nucleus relative to the endothelial cell monolayer. MetaMorph software (Molecular Devices) was used to analyze the imaging data and score TEM, T cell morphology and nuclear positioning. Between experimental repeats, fluorescent dye labeling was swapped between control and MyoIIA KO cells to control for potential effects of the dyes.

### Quantification of T cell crawling on endothelial cells

Time-lapse images of T cells crawling over bEnd.3 endothelial cells and undergoing TEM under shear flow were analyzed using Imaris software (Bitplane). Control and MyoIIA KO T cells were tracked based on their fluorescent label using Imaris’ ‘Spot’ function; from the cell tracks the average speed and displacement over time were calculated for each individual cell.

### Quantification of MyoIIA distribution during TEM

To image the dynamics of MyoIIA localization during TEM we used activated T cells derived from transgenic mice expressing a GFP-MyoIIA fusion protein [17]. *In vitro* activated GFP-MyoIIA T cells were used in TEM under flow assays in which the bEnd.3 endothelial cells were labeled with Alexa647-conjugated CD31 (PECAM-1) antibodies (Biolegend) to visualize the endothelial cell junctions. Z-stack time-lapse images were acquired every 15 sec for 30 min on a spinning-disk confocal microscope. In addition, to determine the distribution of endogenous MyoIIA in WT T cells, we perfused T cells into flow chambers containing endothelial cell monolayers (as described above) and at various time-points we fixed the cells under flow with 3% paraformaldehyde for 10 min. After permeabilizing with Saponin (Sigma) and blocking with normal donkey serum (Jackson ImmunoResearch) for 30 min, the cells were stained with rabbit polyclonal primary antibodies to MyoIIA (BTI). After extensive washing, the cells were stained with FITC-labeled anti-rabbit secondary antibodies (Jackson ImmunoResearch) to detect MyoIIA, Phalloidin-Alexa555 (Invitrogen) to detect polymerized F-actin, and DAPI (Invitrogen) to visualize nuclei. Z-stack images were acquired with a spinning-disk confocal microscope. Image analysis and quantification of MyoIIA distribution was done using MetaMorph software and the ‘Line scan’ function.

### Quantification of T cell morphology and nucleus position during TEM

Images of T cells undergoing TEM acquired 20 min after addition onto endothelial monolayers were analyzed to determine the position of the T cell nucleus (labeled with Hoechst nuclear dye) relative to the endothelial monolayer. The T cell nucleus was scored as being above the endothelial monolayer if it was contained in a portion of the T cell body surrounded by a white phase contrast ring. T cell morphology was analyzed based on the cell body shape determined by the fluorescent dye label. Cells with elongated or multiple protrusions were scored as abnormal. Nucleus positioning and morphology of control and MyoIIA KO T cells were scored blindly.

### Sub-cutaneous tumors

Sub-cutaneous ectopic tumors were induced in WT C57BL/6 mice by sub-cutaneous injection of 5-10x10^5^ EG.7-Ova cells in the hind flanks of recipient mice. Tumor growth was monitored daily and the tumor bearing mice were used for T cell trafficking experiments once tumors reached ≥4mm (typically 10-14 days after injection). Mice with tumors larger than 10mm were immediately euthanized. These procedures were approved and carried out in accordance to the regulations of the Institutional Animal Care and Use Committees of the University of California, San Francisco, and all efforts were made to minimize mouse suffering.

### EAE induction and monitoring

EAE was induced using MOG induction kits from Hooke Laboratories according to their protocol. Briefly, WT C57BL/6 mice of at least 8 weeks of age were immunized with 200μg of MOG_33-55_ peptide emulsified in complete Freund’s adjuvant injected sub-cutaneously, followed by intra-peritoneal injection of 200ng pertussis toxin on the day of induction and the following day. Typical EAE onset was within 13-15 days post immunization. Mice were monitored and scored daily for development of EAE based on the following 0-5 scoring criteria: 0, no disease; 1 limp tail; 2, weakness or partial paralysis of hind limbs; 3, full paralysis of hind limbs; 4, complete hind limb paralysis and partial front limb paralysis; 5, complete paralysis of front and hind limbs or moribund state. Mice with a score higher than 4 were immediately euthanized. Mice with scores of 2 or greater were used for T cell trafficking experiments. These procedures were approved and carried out in accordance to the regulations of the Institutional Animal Care and Use Committees of National Jewish Health, and all efforts were made to minimize mouse suffering.

### *In vivo* T cell trafficking

Sorted GFP^+^ control and MyoIIA KO T cells were over-labeled with either 2-4μM CFSE or 2-4μM CellTrace Far Red DDAO-SE (Invitrogen) and mixed at a 1:1 ratio, and 2-6x10^6^ total T cells were injected intravenously into recipient mice. For homeostatic trafficking in untreated recipient mice, 18h after adoptive transfer, recipient mice were euthanized and lymph nodes, blood and spleen were harvested. After lymph node and spleen dissociation, the number of transferred control and MyoIIA KO T cells was quantified by flow cytometry and the ratio of KO vs. control T cells in the blood, lymph nodes and spleen of recipient mice was determined. The ratios for the lymph node and spleen were normalized to the ratio in the blood to correct for potential differences in the input or survival of control vs. MyoIIA KO T cells.

To quantify T cell trafficking to the site of ectopic sub-cutaneous tumors, a total of 4-8x10^6^ 2-5μM CFSE- or 10-20μM CellTracker Orange CMTMR- labeled control and MyoIIA KO Ova-specific CD8 T cells mixed at a 1:1 ratio were intravenously transferred into EG.7-Ova tumor-bearing recipient mice after the establishment of a sub-cutaneous tumor site in the flank of recipient mice (as described above). 18h after adoptive transfer, recipient mice were euthanized and tumors and blood were harvested and single cell suspensions were obtained. Tumors were digested using collagenase D and DNAse I for 30 min. The number and ratio of transferred control and MyoIIA KO T cells was then quantified by flow cytometry and the ratio for the tumor was normalized to the ratio in the blood.

To measure the effects of MyoIIA KO on self-reactive T cell trafficking to the Central Nervous System, a total of 4-8x10^6^ 1μM Violet Proliferation Dye (VPD) (BD Biosciences)-labeled or 5μM Cell Proliferation Dye eFluor670 (eBiosciences)- labeled control and MyoIIA KO MOG-specific CD4 T cells mixed at a 1:1 ratio were intravenously transferred into recipient mice with overt EAE (score of 2 or greater, as described above). 24h after adoptive transfer, recipient mice were euthanized, spinal cords and blood were harvested, and single cell suspensions were obtained by mechanical dissociation. The number and ratio of transferred control and MyoIIA KO T cells in the spinal cord and blood was quantified by flow cytometry. To correct for potential differences in the input or survival of control vs. MyoIIA KO T cells, the ratio for the spinal cord was normalized to the ratio in the blood.

### Statistical analysis

Prism software (GraphPad) was used to graph the data and calculate statistical significance. The statistical significance of data was determined by performing either Student’s t-test, for single comparisons, or analysis of variance (ANOVA) for multiple comparisons, followed by post hoc Tukey tests. To determine the significance of nominal variable data Fisher’s exact test was used.

## Supporting Information

Movie S1 **MyoIIA deficiency in activated T cells causes defects in trans-endothelial migration (TEM) under flow.** Fluorescently labeled control (green) and MyoIIA KO (red) activated T cells were mixed at a 1:1 ratio and perfused into a flow chamber containing a monolayer of bEnd.3 brain endothelial cells and then kept under physiological shear flow for 20 min. Phase contrast, green and red fluorescence images were acquired every 15 sec. At the end of the movie, yellow arrows point to T cells that completed diapedesis and white arrows point to T cells that have not completed diapedesis. The color-coded tracks show the migration paths of each T cell during the time-lapse. Time in min: sec.
(MOV)

Movie S2 **TEM of a control activated T cell.** Representative TEM under physiological shear flow of a control activated T cell perfused into a flow chamber containing a monolayer of bEnd.3 brain endothelial cells. Phase contrast and fluorescence images were acquired every 15 sec during the time-lapse imaging. The white arrow points to the T cell while above the endothelial monolayer; the arrow becomes black as the T cell completes diapedesis. Time in min: sec.
(MOV)

Movie S3 **Behavior of a MyoIIA KO activated T cell while attempting TEM.** Representative MyoIIA KO activated T cell failing to complete TEM. T cells were perfused into a flow chamber containing a monolayer of bEnd.3 brain endothelial cells and then kept under physiological shear flow for 30 min. Phase contrast and fluorescence images were acquired every 15 sec during the time-lapse imaging. The white arrow points to the body of the T cell which remains above the endothelial monolayer for the duration of the time-lapse. Time in min: sec.
(MOV)

Movie S4 **Control and MyoIIA KO T cell migration over endothelial cells during TEM.** Fluorescently labeled control (green) and MyoIIA KO (red) activated T cells were mixed at a 1:1 ratio and perfused into a flow chamber containing a monolayer of bEnd.3 brain endothelial cells and then kept under physiological shear flow for 15 min. Phase contrast, green and red fluorescence images were acquired every 15 sec. The color-coded tracks show the migration paths of each T cell during the time-lapse. Time in min: sec.
(MOV)

Movie S5 **Uropodal enrichment of MyoIIA during T cell diapedesis.** Control activated T cells expressing a fusion protein of GFP and MyoIIA (green) were imaged by time-lapse confocal microscopy while undergoing TEM under flow over a monolayer of bEnd.3 brain endothelial cells. The endothelial cells were stained with APC-conjugated anti-CD31 (red) to visualize endothelial cell junctions. Green and red fluorescence Z-stack images were acquired every 15 sec during the time-lapse. Maximum Z-projection images of the time-lapse movie are shown. Blue arrows point to the leading-edge of the T cell located under the endothelial cell monolayer; yellow arrows point to the GFP-MyoIIA enrichment as the T cell completes squeezing its back through the endothelial cell monolayer. Time in min: sec.
(MOV)
